# Extracting New Temporal Features to Improve the Interpretability of Undiagnosed Type 2 Diabetes Mellitus Prediction Models

**DOI:** 10.3390/jpm12030368

**Published:** 2022-02-28

**Authors:** Simon Kocbek, Primož Kocbek, Lucija Gosak, Nino Fijačko, Gregor Štiglic

**Affiliations:** 1 Institute of Informatics, Faculty of Electrical Engineering and Computer Science, University of Maribor, 2000 Maribor, Slovenia; gregor.stiglic@um.si; 2 Faculty of Health Sciences, University of Maribor, 2000 Maribor, Slovenia; primoz.kocbek@um.si (P.K.); lucija.gosak2@um.si (L.G.); nino.fijacko@um.si (N.F.); 3 Usher Institute, University of Edinburgh, Edinburgh EH8 9YL, UK

**Keywords:** diabetes mellitus type 2, prediction model, LogicRegression, interpretability

## Abstract

Type 2 diabetes mellitus (T2DM) often results in high morbidity and mortality. In addition, T2DM presents a substantial financial burden for individuals and their families, health systems, and societies. According to studies and reports, globally, the incidence and prevalence of T2DM are increasing rapidly. Several models have been built to predict T2DM onset in the future or detect undiagnosed T2DM in patients. Additional to the performance of such models, their interpretability is crucial for health experts, especially in personalized clinical prediction models. Data collected over 42 months from health check-up examinations and prescribed drugs data repositories of four primary healthcare providers were used in this study. We propose a framework consisting of LogicRegression based feature extraction and Least Absolute Shrinkage and Selection operator based prediction modeling for undiagnosed T2DM prediction. Performance of the models was measured using Area under the ROC curve (AUC) with corresponding confidence intervals. Results show that using LogicRegression based feature extraction resulted in simpler models, which are easier for healthcare experts to interpret, especially in cases with many binary features. Models developed using the proposed framework resulted in an AUC of 0.818 (95% Confidence Interval (CI): 0.812−0.823) that was comparable to more complex models (i.e., models with a larger number of features), where all features were included in prediction model development with the AUC of 0.816 (95% CI: 0.810−0.822). However, the difference in the number of used features was significant. This study proposes a framework for building interpretable models in healthcare that can contribute to higher trust in prediction models from healthcare experts.

## 1. Introduction

Morbidity and mortality are often results of Type 2 diabetes mellitus (T2DM). In addition, T2DM presents a substantial financial drain for individuals and families, health systems, and societies. Globally, the incidence and prevalence of T2DM are increasing rapidly [1]. In 2017, it was estimated that 425 million people had any diabetes (approx. 5.5% of the worldwide population), of which 90% had T2DM. According to projection estimations, the prevalence is going to increase substantially in the coming years; by 2045, for example, a 48% increase of prevalence from the above numbers is expected, or in absolute numbers, an estimated 629 million people (approx. 6.6% of the worldwide population) are expected to be suffering from any diabetes [2]. T2DM can also lead to a substantially increased risk of macrovascular and microvascular disease, especially in inadequate glycemic control [3]. Impaired fasting glucose typically leads to slow progression of T2DM and, more importantly, its symptoms may remain undetected for many years. 

Electronic Health Records (EHR) enable researchers to perform predictive modeling by providing a large amount of data [4] and many links have been found between patient health, the environment, and clinical decisions [5]. Nowadays, data mining techniques are applied to various fields of science, including healthcare and medicine [6]. Usually, techniques such as pattern recognition, disease prediction, and classification are used. Although multiple methods are available to build prediction models, prediction accuracy and data validity are often not realistic for model application in practice. Models usually perform well in specific datasets used to build the prediction models but are frequently not adapted sufficiently well when used on other datasets [7].

There is growing interest in clinical prediction, but models’ interpretation is rarely based on end-user needs [8], and there is a lack of model interpretability techniques [9]. Interpretability of results based on predictive models is crucial in critical areas such as healthcare and is essential for adopting models. People often do not understand predictive models and therefore do not trust them [10]. LogicRegression can be used to improve the interpretability of predictive models.

LogicRegression is an adaptive classification and regression procedure which searches for Boolean (logic) combinations of binary variables that best explain the variability in the outcome [11,12]. LogicRegression looks for logical combinations of binary features. We can explain the variability of the outcome feature and thus reveal the features and interactions related to the response and whether they have predictive capabilities [11].

The purpose of this paper is to use LogicRegression to make final models less complex (i.e., with less features) and the features that appear in the interpretation of predictive models much more understandable. This is also important for health professionals, as they do not have the necessary knowledge to apply prediction models or interpret the results obtained. This is also important from the patient’s point of view and the provision of personalized healthcare. Simple interpretation will make it easier for the patient to understand the operation of the predictive model and outcome. The paper presents an example of using extracted features using Logic Regression to improve the personalized interpretability of the prediction models to the end-users.

## 2. Materials and Methods

### 2.1. Data

EHR data consisted of health check-ups and prescribed drugs data from four Slovenian primary healthcare providers for a period of approximately 3.5 years from 12 December 2014 to 27 July 2018. Data for 21,138 medical records and 114 potential useful features were exported from the healthcare information systems after the on-site anonymization process. Our first step was the removal of features with more than 20% of missing data (73 potential features remain). Since our focus when building prediction models was on the fasting plasma glucose level (FPGL) measurement (mmol/L) and results of Finnish Diabetes Risk Score (FINDRISC) features, which included Age, Gender, BMI, Waist circumference, Active_30_min, Medication, High_BS, Grocer, and Diab_fam we selected cases with all those values present (4086 such cases remained). We next removed (a) cases with more than 50% of the features were not available (4067 cases left), (b) removed all duplicate entries (in cases of multiple patient visits only the most recent visit was included) (3535 cases left), (c) cases not having a previous diabetes diagnosis (3176 cases left) and entries where: (d) FPGL was not reported giving us a total of 3120 records of patient visits were left for development of a prediction model to estimate the risk of undiagnosed T2DM. Data included demographics, questionnaire answers for lifestyle choices, physiological measurements, and prescribed medications for two time periods.

Binary features were created for prescribed drugs and questionnaire responses, which resulted in nine numeric and 161 binary features where specific drug related feature was coded as positive in cases where a patient was prescribed with the specific drug during the last 4 months prior to the visit. The target feature was binary, where positive cases were defined as having FPGL higher than 6.1 mmol/L consisting of 24.71% (*n* = 771) of patient visits.

We imputed the remaining missing values using the MissForest based approach [7], which on average meant features with 12.25% missing values as we initially already removed features with 20% or more missing values. MissForest is used to impute missing values particularly in the case of mixed-type data. It can be used to impute continuous and/or categorical data including complex interactions and nonlinear relations. The summary information of the basic predictive and target features can be seen in Table 1. Please see Table A1 for list of all features used in the experiments.

### 2.2. Experimental Setup

The data were split into 80% to derive five extracted features using Logic Regression [13] and 20% to build and evaluate the final prediction models. 

Finally, we created three datasets with the following features: all numeric and binary (170), all numeric and logic (14), and all numeric, binary, and logic features (175). On each dataset, we built a predictive model separately using the same training data.

The Least Absolute Shrinkage and Selection Operator (LASSO) [13] was used to build prediction models. We repeated each 10-fold cross-validation ten times to estimate the variance in Area under the ROC curve (AUC) that was used as our classification performance metric.

## 3. Results

We split the results in this section into two parts. First, we present selected logic attributes extracted from the dataset for the undiagnosed T2DM prediction use case. Next, we present the performance evaluation of the model.

### 3.1. Feature Extraction Using LogicRegression Approach

To demonstrate the practical example of using LogicRegression based extraction of new features to improve interpretability of the prediction models, we provide the results of the first cross-validation run.

The selected use case resulted in five logic features (Table 2) extracted from the complete set of features.

In Table 3, we list all features that were selected in at least 50% of runs in our experiments with LASSO on the dataset with numeric and logic features, while Table 4 lists features for the dataset with numeric, binary and logic features. Frequency (freq) shows in how many experiment runs each feature appeared in the final set of features.

It can be observed that L1, L2, and L3 were used by prediction models derived from the data in all folds of all evaluation runs. Thus, confirming a high contribution of extracted logic features.

In the case of results from a much wider set of features (Table 4), we can see a higher variance in selection by the final prediction models. Four (L2, L3, L4, L5) logic features can be found among the varaibles that were selected in at least 50% of evaluation runs.

### 3.2. Performance Evaluation 

**Figure 1:**
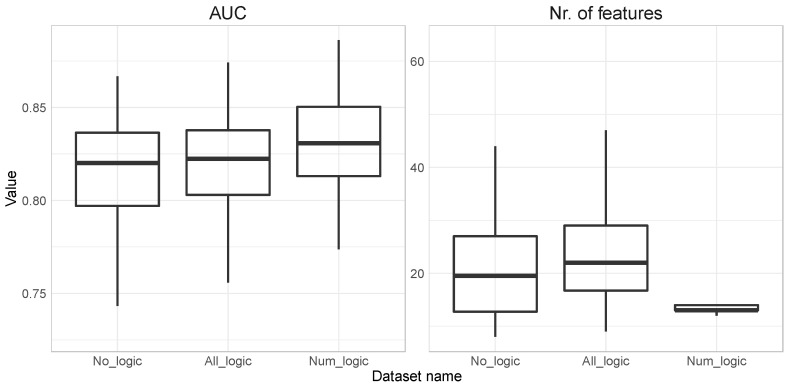
Selected features with the Least Absolute Shrinkage and Selection Operator (LASSO) on the dataset with numeric and logic features.

In Figure 1, we summarize AUC and a selected number of features for all three datasets: no_logic (numeric and binary features), all_logic (numeric, binary, and logic features), and num_logic (numeric and logic features). 

We can observe a slowly increasing average AUC from 0.816 (Standard Deviation (SD)) = 0.03) in no_logic to 0.819 (SD = 0.03) in all_logic and finally 0.829 (SD = 0.03) in the num_logic dataset. When looking at the number of selected feature averages and its variation, we can observe that it slowly increases from 21.7 (SD = 11.18) in no _logic to 23.7 (SD = 10.09) in all_logic but it then almost halves to 13.35 (SD = 0.63) in the num_logic dataset. The SD is steadily increasing in the first two cases, but then it decreases sharply to below 1 (SD = 0.63), which means that out of the 100 repetitions in 92 cases 13 or 14 features were selected in the num_logic dataset. This indicates a very stable final prediction models when comparing num_logic based solutions to no_logic or all_logic.

## 4. Discussion and Conclusions

In this paper, we compared three dimensionality reduction approaches to improve the interpretability of undiagnosed T2DM prediction models (Please note that the calibration of a prediction models was not the scope of this paper and presents a limitation). A simple LASSO regression approach is compared to two variants where a pre-selection of predictive features is conducted on the training set using LogicRegression to consequently simplify a final set of features obtained by the LASSO regression. We kept all original features with added logic features in the first variant, while in the second variant, we kept only numeric and logic features.

Results showed that logic features resulted in simpler models with lower number of features, which are potentially easier to interpret by healthcare experts. This is especially important in the field of personalized medicine. Measured AUC was similar to more complex models, where all features were included. It should be noted that although our method resulted in a lower number of features, some of the logic features may not be straightforward to interpret (e.g., the feature L3 in this paper). To address this issue, we plan to include an interactive system in our future work, where the user would specify the maximum number of original features included in generated logic features in cases where the final model would include many complex logic features. As a result of the current work, in cases when some of the final features are hard to interpret, we recommend that the user uses LogicRegression settings to adjust the complexity of final logic features for achieving satisfactory results.

When healthcare professionals and patients know which features are important in obtaining the outcome of a prediction model and how they can be combined, it helps to understand and increase the level of trust in the decision-making systems [10]. With greater interpretability of the model, we better understand and interpret the forecast for end-users and improve the support in decision-making for health professionals based on data [14]. More complex models such as deep neural networks [15] allow high accuracy but are difficult to explain. Simple models (e.g., decision trees) are less accurate but allow for more straightforward explanations [16]. Therefore, sophisticated machine learning models usually offer better performance than traditional simple models but are difficult for health professionals to understand. However, in many cases simple models also provide good classification performance, which is not significantly different from more complex models [17]. Our results confirm this hypothesis. Comprehensible models are known for their contribution to higher trust in prediction models from the end-users in healthcare.

Interpretability techniques are often categorized according to the time period used to develop the machine learning model [14]. Pre-model approaches are independent of the model and may be employed prior to making a choice on which model to use. Our approach presented in this study belongs in this group of interpretability approaches along with techniques such as Principal Component Analysis (PCA), t-Distributed Stochastic Neighbor Embedding (t-SNE), and some clustering techniques. While Molnar [18] classifies PCA, t-SNE, and clustering methods as interpretable methods, it is worth noting that the interpretability of attributes transformed using PCA, embeddings, or clusters cannot provide comprehensible medical interpretation, but can be used to visualize the results and highlight patterns of interest from an interpretability standpoint. The proposed approach is much more interpretable, despite the possible complex combinations of features that might occur as a result of LogicRegression.

During the experiments, we also observed the unstable behavior of logic regression, where different logic features were selected with each run of the cross-validation. Although this did not influence the average number of selected features it resulted in instability of the interpretability of the model. Another limitation are the combinations of the features used in extracted features. For example, the first extracted feature (L1) suggested that checking whether a person did not experience elevated blood sugar in the past should be accompanied by checking for sulfadiazine and trimethoprim use in the last 4 months – this extracted feature works as a protective factor as seen from Table 3. We see this as a disadvantage of logic regression since different conclusions can be made based on selected features. This could be resolved to some extent by using exhaustive search methods to extract logic features resulting in extremely long running times, presenting another drawback, especially in cases where personalized models would be built. To personalize the solution even further, it would be worth exploring the prediction model development for each specific patient at the time of the examination using the subset of the data where patients similar to the examined patient would be assigned a higher weight in comparison to other patients (boosting principle).

Although our work is the field of healthcare, we believe that our results can also be applied in other emerging fields of applied prediction modeling where interpretability of results is important such as security [19] or ecology [20]. In future work, we will explore effectiveness of our methods in the broader field of security, specifically, to help us understand how misinformation (e.g., intentionally misleading information) is being spread.

## Figures and Tables

**Table 1:** Summary table basic predictive and target features for healthcare centers.

Original Feature Name	Description	FPGL ≤ 6.1 mmol/L [75.29% [*n* = 2349]]	FPGL > 6.1 mmol/L [24.71% [*n* = 771]]
Age [mean (standard deviation − SD)]	Age in years	56.07 (SD = 13.2)	61.77 (SD = 10.98)
Gender_M [%(*n*)]	Percentage of males	37.16 (*n* = 873)	54.47 (*n* = 420)
BMI [mean (SD)]	Body mass index	28.89 (SD = 5.39)	32.16 (SD = 13.21)
WC [mean (SD)]	Waist circumference in cm	96.25 (SD = 13.89)	103.48 (SD = 13.8)
Active_30_min (Q2) [%(*n*)]	Active at least 30 minutes a day?	64.88 (*n* = 1524)	52.27 (*n* = 403)
Medication (Q3) [%(*n*)]	Blood pressure medication?	40.19 (*n* = 944)	60.18 (*n* = 464)
High_BS [%(*n*)] (Q4)	Ever measured high blood sugar?	7.32 (*n* = 172)	47.47 (*n* = 366)
Grocer [%(*n*)] (Q18)	Eat vegetable/fruit daily?	90.59 (*n* = 2128)	78.99 (*n* = 609)
Diab_fam [%(*n*)] (Q6)	Diabetes in family?	69.65 (*n* = 1636)	61.74 (*n* = 476)
FPGL [mean (SD)]	Fasting plasma glucose level	5.26 (SD = 0.44)	6.74 (SD = 0.8)

**Table 2:** Extracted logic features with corresponding LogicRegression rules and descriptions.

Feature	Rule	Description
L1	(ATC_J01EE01 or (not Q41))	Prescribed sulfadiazine and trimethoprim, or never measured high blood sugar.
L2	Q51	Seldom eat fruit and vegetable.
L3	((ATC_M01AE02 and ATC_J01CE10) or (not SE))	Prescribed naproxen and benzathine phenoxymethylpenicillin or not socially endangered.
L4	Q494	Daily consumption of alcohol in the last 12 months.
L5	(MSE or ATC_D01AE15)	Medium socially endangered or prescribed antifungals for dermatological use.

**Table 3:** Selected features with the Least Absolute Shrinkage and Selection Operator (LASSO) on the dataset with numeric and logic features.

Feature	Freq	Description
−Gender	100	Gender
+Blood_pressure	100	Blood pressure
+Heart_beat	100	Heart_beat
+Age	100	Age
+BMI	100	Body mass index
+WC	100	Waist circumference
−L1	100	Logic feature 1
−L2	100	Logic feature 2
−L3	100	Logic feature 3
−Body_height	99	Body height
+Body_weight	83	Body weight

**Table 4:** Selected features with LASSO on the dataset with binary, numeric, and logic features.

Feature	Freq	Description
−L3	100	Logic feature 3
−L4	100	Logic feature 4
+L5	100	Logic feature 5
+Blood_pressure	100	Blood pressure
+WC	100	Waist circumference in cm
+Heart_beat	100	Heart_beat
+Age	100	Age in years
+Q45	100	Ever measured high blood sugar? Yes
−Gender	100	Gender
+Q32	93	Using drug(s) for lowering blood pressure
+Body_weight	87	Body weight
−Non_smoker	87	Non-smoker
+L2	79	Logic attribute 2
−Q321	78	Most often used oil is vegetable oil
−Non_drinker	77	No alcohol consumption
−Q583	75	Handle stress with hardship
+Q62	74	Parent, brother, or sister have diabetes
+BMI	69	Body Mass Index
−Q161	63	2 meals per day on average
−Q301	51	No habit of using salt at the table

## Data Availability

Restrictions apply to the availability of these data. Data was obtained from Nova vizija d.d. and are available from the authors with the permission of Nova vizija d.d.

## Appendix A

**Table A1:** List of original features with description and their possible values. Please note that Nominal features were processed in such a way that for each possible value a new feature was generated. For example, the feature Q43 resulted in three features for each possible value (new features were named Q431, Q432 and Q433). Drug features are marked with the Anatomical Therapeutic Chemical (ATC) classification. The final set contained 170 features.

Name	Description	Value
Age	Age of the patient	Numeric
Gender	Gender of the patient	Male, Female
BMI	Body Mass Index of the patient	Numeric
Blood_pressure	Blood pressure of the patient	Numeric
WC	Waist circumference of the patient	Numeric
Heart_beat	Heart beat of the patient	Numeric
Body_weight	Body weight of the patient	Numeric
Body_height	Body height of the patient	Numeric
Smoking_status	Smoking status of the patient	Non-smoker, Smoker, Ex-smoker, Passive smoker
Eating_habits	Assessment of eating habits	Adequate, Satisfactory, Inadequate
Drinking_status	Drinking status	Abstinent, Less risky drinking, Risky, Harmful, Addictive
SDH	Social determinants of health	Not threatened, Medium threatened, Threatened
PAS	Physical activity status	Sufficient, Borderline, Insufficient
Stress	Level of stress	Not threatened, Threatened
RD	Risk of depression	No significant risk of depression, Risk of depression
Q18	How often do you usually eat vegetables?	Never Points, 4-6 times a week, 1x a day, More than 1x a day
Q16	How many meals do you eat on average per day?	2 or less, 3 to 5, 6 or more
Q2	Are you physically active for at least 30 min/day?	Yes, No
Q3	Do you take medication to lower your blood pressure?	Yes, No
Q30	Do you have a habit of salting dishes at the table?	Yes, No
Q32	On average, which type of fat do you use most in food preparation or as a spread?	Vegetable oils, Cream, Butter, Lard, Hard margarines, Soft margarines, High-fat spreads, Low-fat spreads, Chocolate spread, Peanut butter, Pate, Cream Spread, Mayonnaise
Q4	Have you ever had your blood sugar measured?	Yes, No
Q43	How many times in a typical week do you engage in vigorous physical activity for at least 25 minutes each time to the point where you are breathing and sweating?	0 or 1 times per week, 2 times per week, 3 or more times per week
Q44	How many times in a typical week do you engage in moderate physical activity for at least 30 minutes each time, to the extent that you breathe a little faster and warm up?	0 or 1 times per week, 2 to 4 times per week, 5 or more times per week
Q47	How often have you drunk drinks containing alcohol in the last 12 months?	Never, Once a month or less, 2 to 4 times a month, 2 to 3 times a week, 4 or more times a week
Q48	In the last 12 months, how many measures of a drink containing alcohol did you usually have when you were drinking?	Zero to 1 measure, 2 measures, 3 or 4 measures, 5 or 6 measures, 7 or more
Q49	In the last 12 months, how often have you had 6 or more sips on one occasion for men and 4 or more sips on one occasion for women?	Never, Less than once a month, 1 to 3 times a month, 1 to 3 times a week, Daily or almost daily
Q51	In the last 12 months, how often have you needed an alcoholic drink in the morning to recover from excessive drinking the day before?	Never, Less than once a month, 1 to 3 times a month, 1 to 3 times a week, Daily or almost daily
Q57	How often do you feel tense, stressed or under a lot of pressure?	Never, Rarely, Occasionally, Often, Every day
Q58	How do you manage the tensions, stresses and pressures you experience in your life?	Easily, Able to, Able to with more efforts, Very difficult, Can’t
Q59	How often in the past 2 weeks have you felt little interest and satisfaction in the things you do?	Not at all, A few days, More than half the days, Almost every day
Q6	Does family have diabetes?	No, Outer family, Inner family
Q60	How often have you felt depressed, depressed, despairing in the past 2 weeks?	Not at all, A few days, More than half the days, Almost every day
Q69	Please indicate the last school you attended.	Primary school incomplete, Primary school, 2 or 3-year vocational school, 4-year secondary school or gymnasium, Graduate, Postgraduate
Q70	What is your current employment status?	Employed, Self-employed, Unemployed, Student, Retired, Disabled pensioner, Permanently disabled, Housewife
Q71	How do you get through the month based in income?	Good, Occasional problems, I have problems
ATC_A02BC01	Omeprazole	Binary (0,1)
ATC_A02BC02	Pantoprazole	Binary (0,1)
ATC_A11CC05	Colecalciferol	Binary (0,1)
ATC_B01AC06	Acetylsalicylic acid	Binary (0,1)
ATC_C03BA11	Indapamide	Binary (0,1)
ATC_C07AB07	Bisoprolol	Binary (0,1)
ATC_C09AA04	Perindopril	Binary (0,1)
ATC_D01AC01	Clotrimazole	Binary (0,1)
ATC_D01AE15	Terbinafine	Binary (0,1)
ATC_D07AC13	Mometasone	Binary (0,1)
ATC_G04BD09	Trospium	Binary (0,1)
ATC_J01CA04	Amoxicillin	Binary (0,1)
ATC_J01CE10	Benzathine phenoxymethylpenicillin	Binary (0,1)
ATC_J01CR02	Amoxicillin and beta-lactamase inhibitor	Binary (0,1)
ATC_J01EE01	Sulfadiazine /trimethoprim	Binary (0,1)
ATC_J01FA10	Azithromycin	Binary (0,1)
ATC_M01AB05	Diclofenac	Binary (0,1)
ATC_M01AE01	Ibuprofen	Binary (0,1)
ATC_M01AE02	Naproxen	Binary (0,1)
ATC_N02AJ13	Tramadol and paracetamol	Binary (0,1)
ATC_N02BB02	Metamizole sodium	Binary (0,1)
ATC_N02BE01	Paracetamol	Binary (0,1)
ATC_N05BA08	Bromazepam	Binary (0,1)
ATC_N05BA12	Alprazolam	Binary (0,1)
ATC_N05CF02	Zolpidem	Binary (0,1)
ATC_R01AD09	Mometasone	Binary (0,1)
ATC_R03AC02	Salbutamol	Binary (0,1)
ATC_R03AL01	Fenoterol and ipratropium bromide	Binary (0,1)
ATC_R06AE07	Cetirizine	Binary (0,1)
ATC_R06AX13	Loratadine	Binary (0,1)
ATC_S01AA12	Tobramycin	Binary (0,1)
